# Platelet‐derived growth factor (PDGF)‐BB protects dopaminergic neurons via activation of Akt/ERK/CREB pathways to upregulate tyrosine hydroxylase

**DOI:** 10.1111/cns.13708

**Published:** 2021-08-04

**Authors:** Huan Chen, Yan Teng, Xingmin Chen, Zhihao Liu, Fan Geng, Yanzhuo Liu, Haisong Jiang, Ziyan Wang, Lu Yang

**Affiliations:** ^1^ Institute of Neurology Sichuan Provincial People's Hospital University of Electronic Science and Technology of China Chengdu Sichuan China; ^2^ School of Medicine University of Electronic Science and Technology of China Chengdu Sichuan China

**Keywords:** dopaminergic neuron, Parkinson's disease, PDGF‐BB, tyrosine hydroxylase

## Abstract

**Aims:**

The neurotropic growth factor PDGF‐BB was shown to have vital neurorestorative functions in various animal models of Parkinson's disease (PD). Previous studies indicated that the regenerative property of PDGF‐BB contributes to the increased intensity of tyrosine hydroxylase (TH) fibers in vivo. However, whether PDGF‐BB directly modulates the expression of TH, and the underlying mechanism is still unknown. We will carefully examine this in our current study.

**Method:**

MPTP‐lesion mice received PDGF‐BB treatment via intracerebroventricular (i.c.v) administration, and the expression of TH in different brain regions was assessed by RT‐PCR, Western blot, and immunohistochemistry staining. The molecular mechanisms of PDGF‐BB‐mediated TH upregulation were examined by RT‐PCR, Western blot, ChIP assay, luciferase reporter assay, and immunocytochemistry.

**Results:**

We validated a reversal expression of TH in MPTP‐lesion mice upon i.c.v administration of PDGF‐BB for seven days. Similar effects of PDGF‐BB‐mediated TH upregulation were also observed in MPP^+^‐treated primary neuronal culture and dopaminergic neuronal cell line SH‐SY5Y cells. We next demonstrated that PDGF‐BB rapidly activated the pro‐survival PI3K/Akt and MAPK/ERK signaling pathways, as well as the downstream CREB in SH‐SY5Y cells. We further confirmed the significant induction of p‐CREB in PDGF‐BB‐treated animals in vivo. Using a genetic approach, we demonstrated that the transcription factor CREB is critical for PDGF‐BB‐mediated TH expression. The activation and nucleus translocation of CREB were promoted in PDGF‐BB‐treated SH‐SY5Y cells, and the enrichment of CREB on the promoter region of TH gene was also increased upon PDGF‐BB treatment.

**Conclusion:**

Our data demonstrated that PDGF‐BB directly regulated the expression of TH via activating the downstream Akt/ERK/CREB signaling pathways. Our finding will further support the therapeutic potential of PDGF‐BB in PD, and provide the possibility that targeting PDGF signaling can be harnessed as an adjunctive therapy in PD in the future.

## Graphical abstract

The binding of PDGF‐BB to its receptor in DA neurons leads to the activation of downstream PI3K/Akt and MAPK/ERK signaling pathways, which trigger the nucleus translocation of the transcription factor CREB and increased expression of tyrosine hydroxylase.

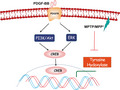

## 1 INTRODUCTION

Parkinson's disease (PD) is a progressive neurodegenerative disease characterized by movement disorder. As patients progressively lose the dopaminergic neurons in the substantia nigra (SN), a dramatic reduction in dopamine (DA) levels in the corpus striatum leads to the clinical phenotype principally presented as resting tremors, rigidity, and bradykinesia.^1^ Based on the symptoms that are caused by loss of DA, “symptomatic therapy” such as intracerebral DA supplementation, or stimulation of dopamine receptors remain the mainstay of treatment for PD. However, those current clinical treatments do not show any recovery function on the particular dopamine‐producing neurons.^2^ Importantly, postmortem tissue analysis of PD patients at different stages illustrated that the number of dopaminergic neurons remains at a level of 50% in the SN after first diagnosis of PD.^3^, ^4^ This observation indicates that, instead of cellular death observed in PD, these cells may remain salvageable. If injured neurons could be supported with certain neuroprotective drugs, those cells could have potential to exert normal function on neurotransmission.^5^, ^6^ Therefore, the identification of efficacious therapeutic agents to improve the function of dopaminergic neurons or compensate for the loss of dopaminergic neurons could slow down or even reverse the neurodegenerative process in PD.

Tyrosine hydroxylase (TH) is a rate‐limiting enzyme in dopamine biosynthesis, which catalyzes the conversion of L‐tyrosine to L‐DOPA, which is a precursor for dopamine. As a marker for dopaminergic neurons in the central nervous system (CNS), TH are predominately expressed in those cells and are responsible for maintaining the level of DA in the brain.^7^, ^8^ Although studies have revealed the regulatory role of TH on DA production, so far, there is no effective medicine to target dopaminergic neurons and improve the expression of TH. As an important endogenous growth factor, the homodimer of the platelet‐derived growth factor isoform B (PDGF‐BB) has been shown to have restorative effects in the dopaminergic system in vivo. In several classic toxin‐induced PD models, it has been shown that intracerebroventricular (i.c.v) application of PDGF‐BB for two weeks not only restored DA neurotransmission, but also provided functional recovery of those animals.^9^, ^10^, ^11^ Importantly, an increased number of nigral TH^+^ cells, as well as the density of the TH^+^ fiber, along with the partial restoration of DA transporter levels, were also detected in PDGF‐BB‐treated animal models.^4^, ^10^ In vitro studies also indicated that the specific effects of PDGF‐BB‐mediated TH expression in dopaminergic neurons are promising.^12^ However, the molecular mechanism leading to this compelling effect is still unknown. Recently, we have demonstrated that PDGF‐BB protects SH‐SY5Y cells from neurotoxin MPP^+^‐induced ROS generation and cellular loss.^13^ Implicating activation of those pro‐survival signaling pathways could be beneficial to the normal function of dopaminergic neurons, such as TH expression.

In this study, we evaluated the effect of PDGF‐BB on TH expression in vivo by using the MPTP‐lesioned mice model. We also tested the effect of PDGF‐BB on TH expression in both primary dopaminergic neuron and dopaminergic neuronal cell line SH‐SY5Y cells. Here, we provide evidence to show that the signaling pathways PI3K/Akt, as well as MAPK/ERK, are activated and play an important role in this process. We have also demonstrated that PDGF‐BB promotes the expression of TH via triggering the nucleus translocation of CREB, which acts as a transcriptional activator of TH genes in dopaminergic neuronal cells. Our data further evaluated the prominent therapeutic potential of PDGF‐BB and the molecular mechanism of PDGF signaling in the dopaminergic system.

## 2 MATERIALS AND METHODS

### 2.1 Chemical reagents and antibodies

MPTP (1‐Methyl‐4‐phenyl‐1,2,3,6‐tetrahydropyridine) and MPP^+^ (1‐methyl‐4‐phenylpyridine) were purchased from Sigma‐Aldrich (Cat#M0896; D048). Human PDGF‐BB was obtained from Genscript (Cat#Z02892). STI‐571 [4‐[(4‐methylpiperazin‐1‐yl) methyl]‐N‐ [4‐methyl‐3‐ [(4‐pyridin‐3‐ylpyrimidin‐2‐yl) amino] phenyl] benzamide], LY294002 [2‐(4‐morpholinyl) ‐8‐phenyl‐1(4H)‐benzopyran‐4‐one], and U0126‐EtOH [1,4‐diamino‐2, 3‐dicyano‐1, 4‐bis (o‐aminophenylmercapto) butadiene] were purchased from Selleckchem (Cat#152459‐95‐5; 154447‐36‐6, and 1173097‐76‐1). Primary antibodies for phospho‐Akt (Cat#p2717), Akt (Cat#9272s), phospho‐ERK (Cat#4374s), ERK (Cat#9107s), phospho‐CREB (Cat#9198s), and CREB (Cat#9197s) were from Cell signaling technology (CST). Anti‐tyrosine hydroxylase (Cat#25859‐1‐AP) and anti‐β‐actin (Cat#60008) primary antibodies were from Proteintech Group. Anti‐mouse IgG (Cat#7076P2) and anti‐rabbit IgG (Cat#7074P2) secondary antibodies were from CST. Anti‐mouse NeuN (Cat#ab104224) were purchased from Abcam. Alexa Fluor 568‐conjugated goat anti‐rabbit secondary antibody (Cat#A11005) and Alexa Fluor 488‐conjugated goat anti‐mouse secondary antibody (Cat#A11001) were from Thermo Fisher Scientific.

### 2.2 Animals

Pregnant C57BL/6J mice were purchased from Chengdu Dossy experimental animals company. All of the animals were housed under conditions of constant temperature and humidity on a 12‐h light and 12‐h dark cycle. All animal procedures were performed according to the protocols approved by the Institutional Animal Care and Use Committee of the School of Medicine, University of Electronic Science and Technology of China.

### 2.3 PD animal model and intracerebroventricular (i.c.v.) administration

We used 18 eight‐week‐old C57BL/6J mice for three groups in this study, and each group contains six animals. To generate the PD mouse model, mice were injected with MPTP intraperitoneally at the dose of 30 mg/kg/day for five days. Since a PD model would exhibit a reduced motor function, the rotarod test was used to evaluate the successful establishment of a PD model five days after the last injection. For PDGF‐BB administration, we performed intraventricular infusion via an intracerebroventricular catheter at a dose of 50 ng/day for seven days. Three weeks later, the mice were sacrificed, and the brain tissues were dissected, fixed, and collected.

### 2.4 Primary neurons and SH‐SY5Y cell lines culture

Mouse midbrain neurons were prepared and cultured as previously described.^14^ In short, midbrain was isolated from mouse embryos at E16, tissues were digested by trypsin‐EDTA (Gibco, 25200) and supplemented with DNAase (Solarbio, 310D031). Cells were then pelleted by centrifugation and resuspended in a DMEM/F12 medium supplemented with 10% FBS (ZETA, Z7186FBS‐500), 1 × GlutaMAX™‐I (Gibco, 35050), 1% Penicillin‐streptomycin antibiotics (Solarbio, P1400), and 0.5% glucose. Cell number was counted, and cells were plated in 24‐ or 6‐well plates coated with poly‐L‐ornithine using Neurobasal^®^ Medium supplement with 2% B27 (Gibco, 17504). DMEM/F12 medium was changed to Neurobasal medium (Gibco, 21103) and supplemented with 10% FBS, 1 × GlutaMAXTM‐I, 1 × B27 supplement, and 1% penicillin‐streptomycin antibiotics after 24 h. Primary neurons were cultured for at least 10 days to allow normal development and elaboration of neurites. Human neuroblastoma SH‐SY5Y cell line was normally cultured in DMEM/F12 medium supplemented with 10% FBS and 1% penicillin‐streptomycin antibiotics at 37°C in a 5% CO_2_ incubator.

### 2.5 Real‐time quantitative PCR (RT‐qPCR)

Total RNA was extracted by Trizol reagent (Invitrogen). 1 µg of RNA was used for cDNA synthesis according to the manufacturer's instructions (Vazyme, Cat#R323‐01). The synthetic cDNA was amplified by using a SYBER green PCR mix (Vazyme, Cat#Q711‐02/03), mouse TH primers (forward: 5′‐GAC AGT CCT CAC ACC ATC CG‐3′, reverse: 5′‐ CTG TGG GTG GTA CCC TAT GC‐3′), and mouse GAPDH primers (forward: 5′‐CTA CAC TGA GGA CCA GGT TGT C‐3′, reverse: 5′‐GTT ATT ATG GGG GTC TGG GAT GG‐3′). Quantitative analyses of mRNA were conducted using ABI 7500 Fast Real‐Time PCR system (Applied Biosystems).

### 2.6 Western blotting (WB)

SH‐SY5Y cells or brain tissues were lysed in the RIPA buffer (Solarbio, Cat#R0010) and supplemented with protease inhibitor and phosphatase inhibitor (Roche, Cat#04693132001; 04906837001). The concentration of protein was quantified by a BCA kit (Solarbio, Cat#CA1210). A loading buffer was added in protein samples and boiled at 98°C for 5 min. Equal amounts of protein samples were electrophoresed in a sodium dodecyl sulfate‐polyacrylamide gel (10%–12.5%), followed by transferring to PVDF membranes (Millipore). PVDF membranes were blocked with 5% non‐fat milk (BD Biosciences, Cat#232100) or 5% bovine serum albumin (Amresco, Cat#0332) for 1 h at room temperature, and the blots were probed with primary antibodies and secondary antibodies. The blots were detected by an HRP chemiluminescence kit (Millipore, Cat#WBKLS0100) under chemiluminescence imaging analysis system (Tanon, 5200). Quantification of the blots was assessed by Image J.

### 2.7 Immunostaining

Primary neurons were plated on cover slips in a 24‐well plate. After the treatment, the cells were fixed with 4% paraformaldehyde and permeabilized with 0.1% Triton X‐100 in PBS. Cells were then blocked in 10% goat serum for 1 h at 4°C and incubated with TH antibody (1:200) and/or NeuN (1:200) overnight at 4°C, followed by incubation with Alexa Fluor 594‐conjugated goat anti‐rabbit IgG and Alexa Fluor488‐conjugated goat anti‐mouse IgG for 1 h. Fluorescent images were acquired by a fluorescence inversion microscope system (Zeiss).

For immunohistochemistry, mice were perfused transcardially using chilled 4% paraformaldehyde. Free‐floating sections encompassing the entire brain were sectioned at 20 µm using a cryostat. Sections were blocked with 10% normal goat serum (NGS) for 1 h at RT. For immunostaining of TH, tissues sections were incubated with an anti‐TH primary antibody (1:100) at 4°C overnight. Fluorescent‐tagged goat anti‐rabbit (594nm, Red) secondary antibodies were used to probe TH expression, respectively. Immunostained floating tissue samples were gelatin mounted and examined for TH‐positive cells, and images were captured at wavelengths encompassing the emission spectra of the probes with a 40× objective. For DAB staining, the sections were then incubated with a biotin‐labeled secondary antibody (Southern Biotechnology Associates) at a 1:500 dilution for 2 h at room temperature (RT) followed by incubation with streptavidin‐conjugated horseradish peroxidase (HRP; Southern Biotechnology Associates) at a 1:500 dilution for 1 h at RT. HRP‐labeled areas were detected using a solution containing 0.3 mg/ml of 3‐3′‐diaminobenzidine (DAB) in 100 mM Tris, pH 7.4, and 0.02% hydrogen peroxide (H_2_O_2_). Sections were examined under bright‐field light microscopy, and images were captured with a 40× objective.

### 2.8 Chromatin immunoprecipitation (ChIP) assay

Cells were cultured in 10 cm dishes and treated (or untreated) with PDGF‐BB (50 ng/ml) for 2 h at a density of 80%–90%. Untreated and treated cells were then operated according to the manufacturer's instructions of SimpleChIP^®^ Enzymatic Chromatin IP Kit (Agarose Bead, CST, 9002). Genome DNA was acquired after cross‐link, nuclear processing, and fragmentation. Fragmented DNA was immunoprecipitated by incubation with anti‐p‐CREB antibody overnight at 4°C. Finally, p‐CREB enrichment efficiency was analyzed by PCR assay. Primers targeted to TH gene promoter (forward: 5′‐ GCTGGGGAGTGAAGGCAAT‐3′, reverse: 5′‐CATGGCTCAGTGTGGAGGTC‐3′) were used for PCR.

### 2.9 Dual‐luciferase reporter assay

The oligonucleotide fragment of TH promoter region was amplified from the genomic DNA of SH‐SY5Y cells and inserted into the pGL3‐basic luciferase plasmid (Promega) by using In‐Fusion cloning technology (Takara). SH‐SY5Y cells were transfected with TH promoter region‐inserted pGL3‐basic luciferase plasmid expressed firefly luciferase enzyme, as well as a pRL‐SV40 plasmid‐encoded renilla luciferase enzyme by using Lipofectamine™ 2000 Transfection Reagent (Invitrogen). Forty‐eight hours later, cells were treated (or untreated) with PDGF‐BB (50 ng/ml) for 2 h. The activity of luciferase enzymes was analyzed by using a dual‐luciferin report detection kit according to the manufacturer's instructions (TransDetect^®^, FR201‐01). The transcriptional activities were acquired by calculating the ratio of firefly luciferase activity vs. renilla luciferase activity.

### 2.10 Statistical analysis

All the data are expressed as mean of ±SD. Statistical significance was evaluated by unpaired student's *t*‐test for two groups or one‐way ANOVA followed by Bonferroni for multiple comparisons. All experiments were repeated at least three times, and *p *< 0.05 was considered statistically significant. The experimental data were statistically analyzed and graphed by using GraphPad Software. Before statistical analysis, the data were tested for Gaussian distribution assessed using D'Agostino‐Pearson test. Furthermore, the data failed to show Gaussian distribution has been assessed by nonparametric tests. *p* values <0.05 were considered statistically significant.

## 3 RESULTS

### 3.1 PDGF‐BB increases the expression of TH in MPTP‐lesioned mice model in vivo

**FIGURE 1:**
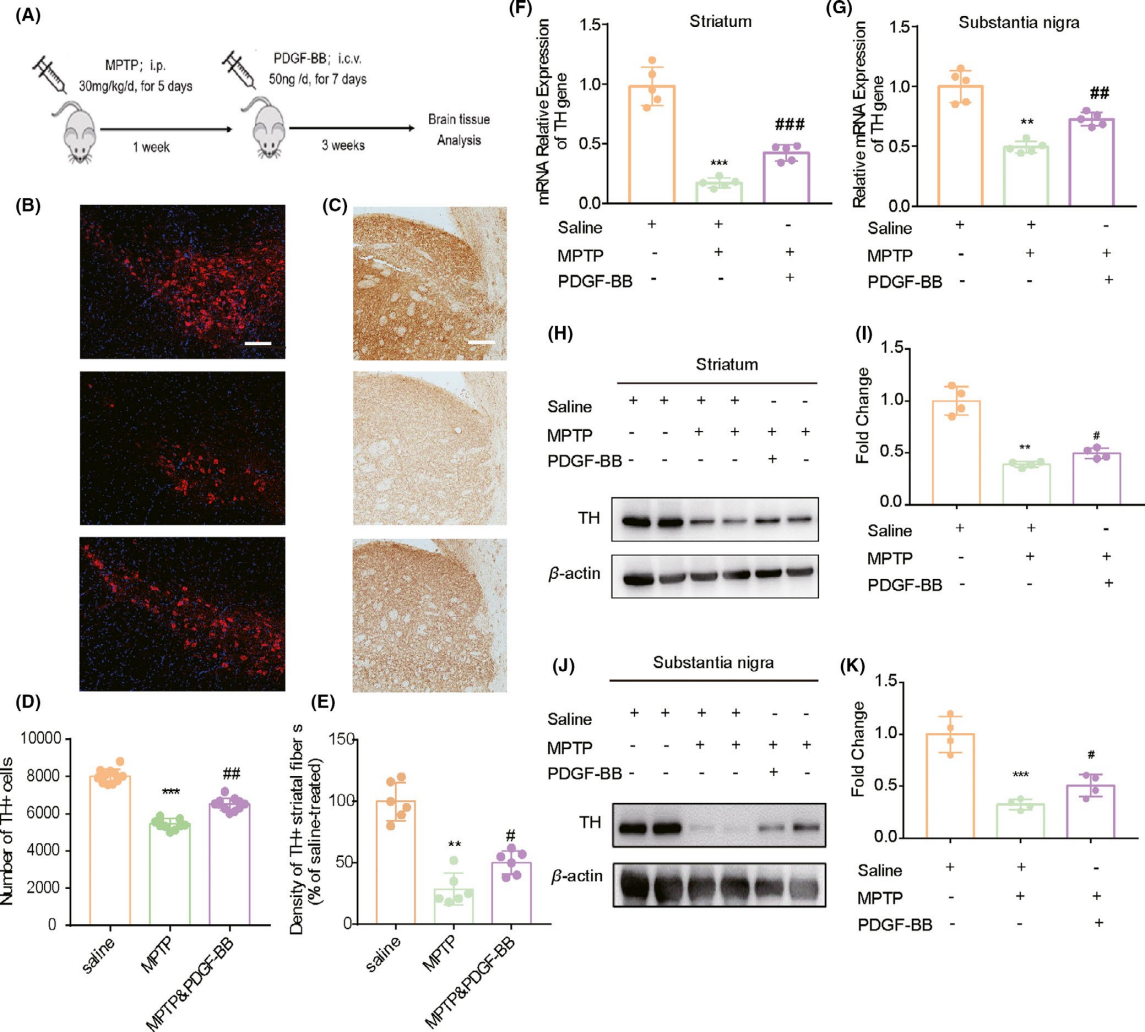
PDGF‐BB‐mediated reversal effect of TH expression in MPTP‐lesioned mice model. (A) Schematic diagram of the animal experiment. (B) Representative images after immunostaining for TH showed the significant reduction in TH‐positive cells in the SN region and (C) the TH‐positive fibers in the striatum of the brain sections from MPTP‐lesioned mice compared with saline‐treated control group. However, this reduction was largely accomplished upon PDGF‐BB treatment. Scale bar: 20 µm. (D) Quantification of numbers of TH^+^ neurons in the SNpc. (E) Ratio of TH^+^ fiber density in the striatum compared with control group. (F) qPCR validation of the TH mRNA level in three experimental groups at the striatum region and (G) the SNpc region. (H–K) Western blotting demonstrated that MPTP injection resulted in a significant decrease in TH expression in brain homogenates, and this effect was partially reversed in MPTP‐lesioned mice administrated with PDGF‐BB. *p < 0.05, **p < 0.01, ***p < 0.001 vs. saline group; #*p *< 0.05, ##*p *< 0.01 vs. MPTP‐treated group

In our study, we decided to first investigate the effect of PDGF‐BB in restoring the expression of TH in the MPTP‐lesioned mice model. We chose MPTP to generate a PD model since it is known as the only neurotoxin, which causes degeneration of dopaminergic neurons in humans diagnosed with PD. Additionally, it rapidly induces degeneration of dopaminergic cell bodies and fibers in the substantia nigra pars compacta (SNpc) and striatum. Mice were treated with PDGF‐BB or vehicle solution for seven days as shown in Figure 1A. Two weeks after the final i.c.v application of PDGF‐BB, mice were sacrificed for examination of the TH expression by Western blot, and immunohistochemistry. As shown in Figure 1B,C, we detected a substantial difference in the number of TH‐immunoreactive neurons in the SNpc and the intensity of TH‐immunoreactive fibers in the dorsal striatum between saline and MPTP‐treated groups. These results demonstrated that MPTP administration led to a dramatic degeneration of nerve fibers in the striatum and the dopaminergic neurons in the SNpc. However, the magnitude of decrease in the TH‐immunoreactive intensity was much lower in the MPTP‐lesioned mice treated with PDGF‐BB compared with the non‐treatment group (Figure 1D,E). We also detected the protein level of TH in tissues collected from different experimental groups. According to our results, both the mRNA level (Figure 1F,G) and the protein level (Figure 1H–K) of TH was reduced in the striatum and the SNpc of MPTP‐treated animals compared with saline‐treated animals, and the restorative effect on TH expression was detected in PDGF‐BB‐treated MPTP group. Thus, consistent with the previous report,^3^ PDGF‐BB administration has a prominent effect in reserving the dopaminergic neurons in the SNpc and increasing the TH^+^ nerve fibers in the striatum in the PD mouse model.

### 3.2 PDGF‐BB induces the expression of TH in dopaminergic neuronal cells in the presence of neurotoxin MPP^+^

**FIGURE 2:**
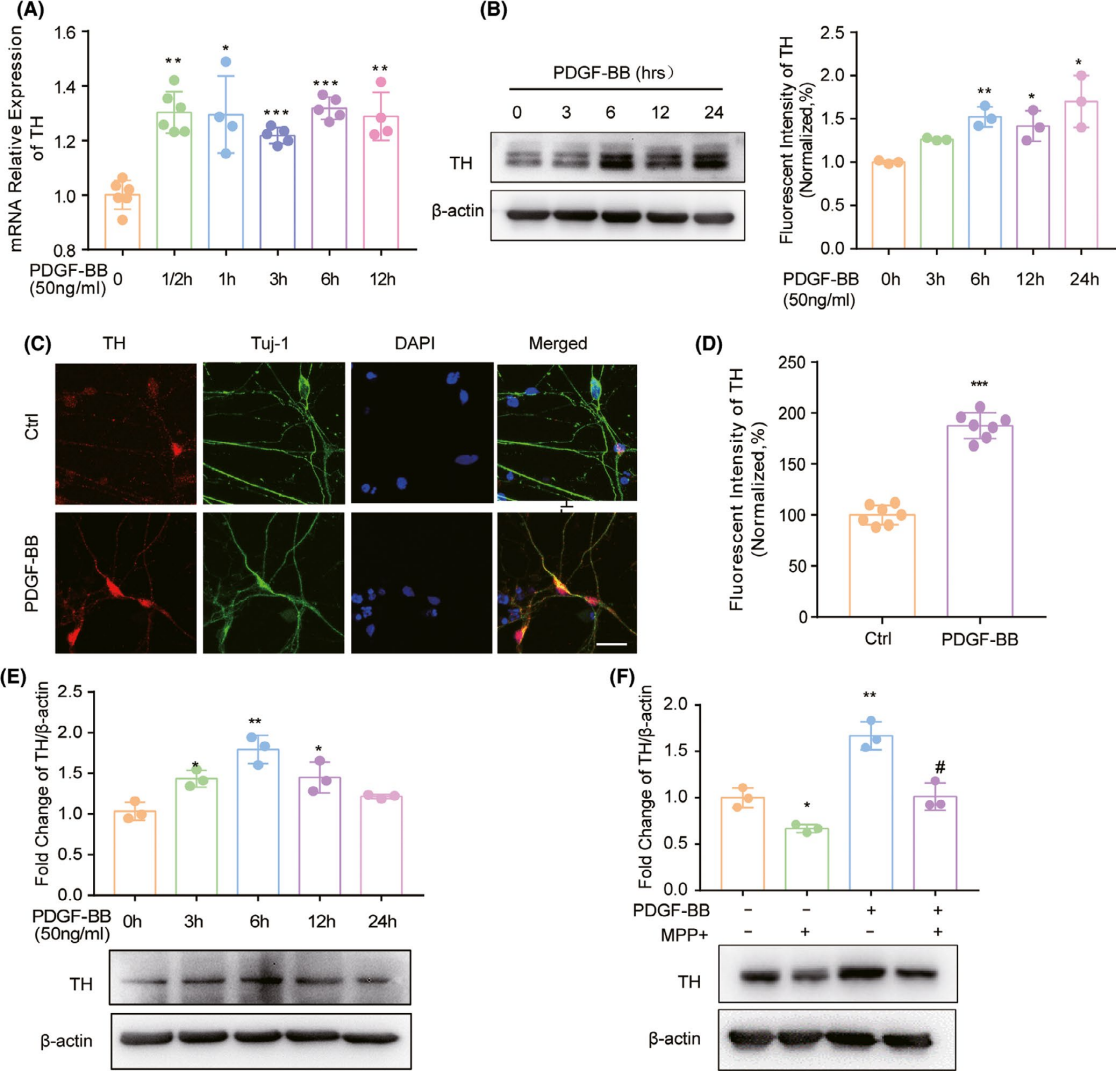
PDGF‐BB‐mediated TH induction in dopaminergic neurons and the in vitro model of PD. (A) Time course of PDGF‐BB‐mediated induction of TH mRNA expression by real‐time PCR in primary dopaminergic neurons. (B) Time course of PDGF‐BB‐mediated induction of TH expression in primary dopaminergic neurons. (C) Representative image of TH and NeuN co‐staining in control or PDGF‐BB‐treated primary dopaminergic neurons and (D) the relative fluorescent intensity. Scale bar: 20 µm. (E) Time course of PDGF‐BB‐mediated induction of TH protein expression by WB analysis in SH‐SY5Y cells. (F) PDGF‐BB application reverse neurotoxin MPP+‐impaired TH expression in SH‐SY5Y cells. All data are presented as mean ± SD of at least three individual experiments. *p < 0.05, **p < 0.01, ***p < 0.001 vs. control group. #*p *< 0.05 vs. MPP^+^ group

From the in vivo study, we confirmed that i.c.v administration of PDGF‐BB promoted the expression of TH in the MPTP‐lesion PD mice model. Next, we wanted to explore the regulatory effect of PDGF‐BB on TH expression in vitro. To do this, we cultured mouse primary dopaminergic neurons and treated cells with PDGF‐BB in a time‐dependent manner using a dose of 50 ng/ml, which was shown to effectively protect neuronal cells against MPP^+^ toxicity in our previous study.^13^ To examine the effect of PDGF‐BB on the expression of TH at both mRNA and protein levels, cells were harvested at indicated time points, respectively. As shown in Figure 2A, the increased transcription of TH gene was observed as early as 30 min after PDGF‐BB exposure, which was evidenced by RT‐PCR. We also detected that PDGF‐BB significantly promoted the expression of TH in primary dopaminergic neurons as shown in Figure 2B, and this effect was confirmed by immunocytochemistry. As shown in Figure 2C,D, PDGF‐BB exposure enhanced the fluorescent intensity of TH in cultured primary dopaminergic neurons. We further verified this effect by using dopaminergic neuronal cell line SH‐SY5Y cells. Similarly, PDGF‐BB treatment lead to a time‐dependent upregulation of TH in SH‐SY5Y cells (Figure 2E). Following this, we wanted to detect the restorative function of PDGF‐BB on TH expression in neurotoxin MPP^+^ exposed SH‐SY5Y cells, which is a widely used cellular model to study the molecular events related to dopaminergic neuronal loss observed in PD. We pretreated SH‐SY5Y cells with 250 µM MPP^+^ for 12 h, followed by the addition of 50 ng/ml PDGF‐BB in the culture medium for another 24 h. Cells were collected, and the expression of TH was detected by WB. As shown in Figure 2F, exposure of MPP^+^ significantly reduced the expression of TH in SH‐SY5Y cells, while PDGF‐BB treatment was able to reverse MPP^+^ caused reduction in TH. Thus, we further confirmed that PDGF‐BB is able to upregulate TH expression in MPP^+^ injured dopaminergic neuronal cells.

### 3.3 Activation of ERK/Akt signal pathways are important for PDGF‐BB‐mediated TH expression

**FIGURE 3:**
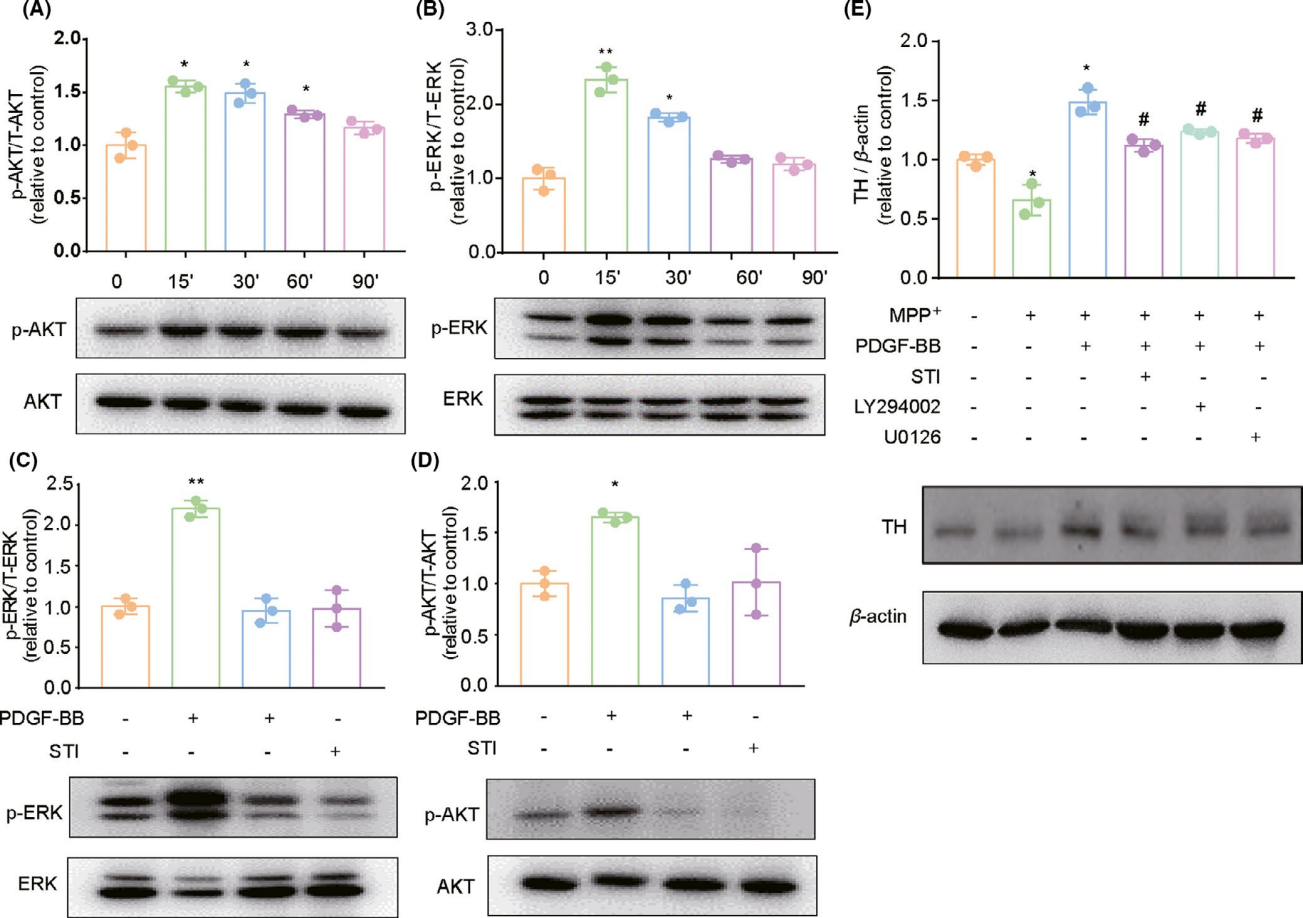
PI3K/Akt and ERK pathways are activated upon PDGF‐BB treatment in dopaminergic neuronal cells. (A) Treatment of SH‐SY5Y cells with PDGF‐BB resulted in increased phosphorylation of AKT, and (B) ERK kinases. (C) Pretreatment of cells with inhibitor of PDGF‐R STI‐571 (5 µM) abrogated PDGF‐BB‐mediated phosphorylation of AKT as well as (D) ERK kinase. (E) Pretreatment of cells with Akt pathway inhibitor LY294002 (5 µM), ERK pathway inhibitor U0126 (10 µM) and STI ‐571 (10 µM) partially abolished PDGF‐BB‐mediated reversal of TH expression in the presence of MPP^+^ in SH‐SY5Y cells. All data are presented as mean ± SD of at least three individual experiments. **p < 0.01 vs. control group; #*p *< 0.05 vs. MPP^+^ treated group

Our next step was to explore the underlying molecular mechanism of PDGF‐BB‐mediated upregulation of TH expression in SH‐SY5Y cells. Based on existing literature and our previous studies, it has been shown that PDGF‐BB mainly activates two intracellular signaling pathways including PI3K/Akt and mitogen‐activated protein kinase (MAPK)‐pathway through activation of its cognitive receptor PDGF‐R in target cells. Our previous study has also demonstrated that upon PDGF‐BB stimulation, PDGFRβ is rapidly activated in both SH‐SY5Y cells and primary dopaminergic neurons (Figure S1).^13^ Therefore, we first validated the activation PI3K/Akt and ERK pathways upon PDGF‐BB treatment in SH‐SY5Y cells. As shown in Figure 3A, we detected a time‐dependent increase in phosphorylated Akt after PDGF‐BB stimulation in SH‐SY5Y cells. We also demonstrated the prominent activation of MAPK/ERK signaling upon PDGF‐BB treatment (Figure 3B), which was evidenced by increased phosphorylation of ERK in SH‐SY5Y cells compared with the level in untreated cells. Since PDGF‐R are the known surface receptors for PDGF‐BB on target cells, by pretreated cells with Imatinib (STI‐571), a pharmacological inhibitor of tyrosine kinase including PDGF receptor, PDGF‐BB‐mediated activation of ERK and Akt signaling were significantly blocked as shown in Figure 3C,D. To further confirm that the effect of PDGF‐BB on PI3K/Akt and ERK activation is related to increased expression of TH, the LY294002 (PI3K pathway‐specific inhibitor) and U0126 (ERK pathway‐specific inhibitor) were used in MPP^+^‐exposed SH‐SY5Y cells. According to our results, it was very obvious that the PDGF‐BB‐mediated reversal effect of TH expression in MPP^+^ treated SH‐SY5Y cells was significantly inhibited upon pretreating cells with either the PDGF‐R inhibitor STI‐571 or the PI3k/Akt and ERK pathways inhibitors, respectively (Figure 3E). Thus, this data indicated that activation of the PI3K/Akt and MAPK/ERK pathways through its cognitive receptor PDGF‐R in SH‐SY5Y cells is essential for PDGF‐BB‐mediated upregulation of TH.

### 3.4 PDGF‐BB mediates activation of downstream transcription factor CREB in dopaminergic neuronal cells

**FIGURE 4:**
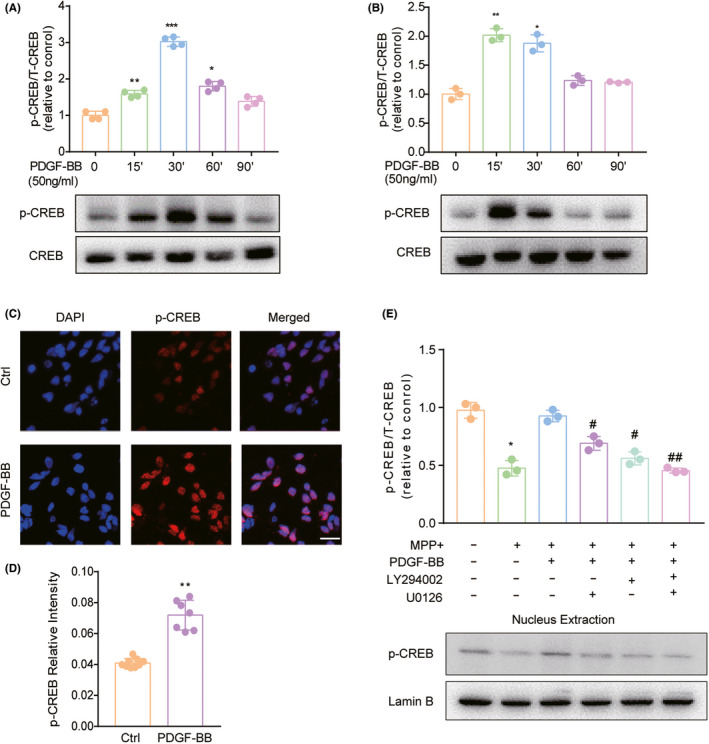
CREB was activated in PDGF‐BB‐exposed dopaminergic neuronal cells. PDGF‐BB‐induced time‐dependent activation of CREB expression in lysates from (A) primary dopaminergic neurons and (B) SH‐SY5Y cells. (C) Representative images of p‐CREB staining and (D) its relative intensity in SH‐SY5Y cells. Scale bars: 10 µm (E) Western blotting demonstrated that inhibitor of Akt and ERK pathways in SH‐SY5Y cells dampened PDGF‐BB‐mediated activation of CREB in the presence of MPP^+^ in SH‐SY5Y cells. All data are presented as mean ± SD of at least three individual experiments. *p < 0.05, **p < 0.01, ***p < 0.001 vs. control group, #*p *< 0.05, ##*p *< 0.01 vs. PDGF‐BB‐treated group

To further explore the downstream transcription factors that are responsible for PDGF‐BB‐mediated upregulation of TH, we analyzed the promoter region of the TH gene and found the presence of a consensus cAMP response element (CRE), which is responsible for CREB activity. CREB, a transcription factor closely related to cell proliferation and survival, has been shown to be the downstream transcription factor upon activation of PI3K/Akt and/or MAPK signaling pathways in previous studies. To explore the role of CREB in PDGF‐BB‐mediated upregulation of TH, primary dopaminergic neurons, and SH‐SY5Y cells were pretreated with PDGF‐BB and assessed for the level of p‐CREB, respectively. As shown in Figure 4A,B, PDGF‐BB treatment triggered rapid phosphorylation of CREB, which was evidenced by WB. We further examined the location of p‐CREB by immunocytochemistry. We also found that upon PDGF‐BB stimulation, a significant increase in fluorescent intensity in the nucleus was detected in PDGF‐BB‐treated SH‐SY5Y cells as shown in Figure 4C,D. By performing further studies using the upstream Akt pathway inhibitor LY294002 and ERK inhibitor U0126, we demonstrated that PDGF‐BB‐mediated activation of CREB in SH‐SY5Y cells through activating PI3K/Akt and ERK signaling pathways. As shown in Figure 4E, pretreated cells with these two inhibitors significantly ameliorate PDGF‐BB‐mediated upregulation of p‐CREB in the nucleus in SH‐SY5Y cells.

### 3.5 PDGF‐BB induces the recruitment of CREB to the promoters of TH gene in dopaminergic neuronal cells

**FIGURE 5:**
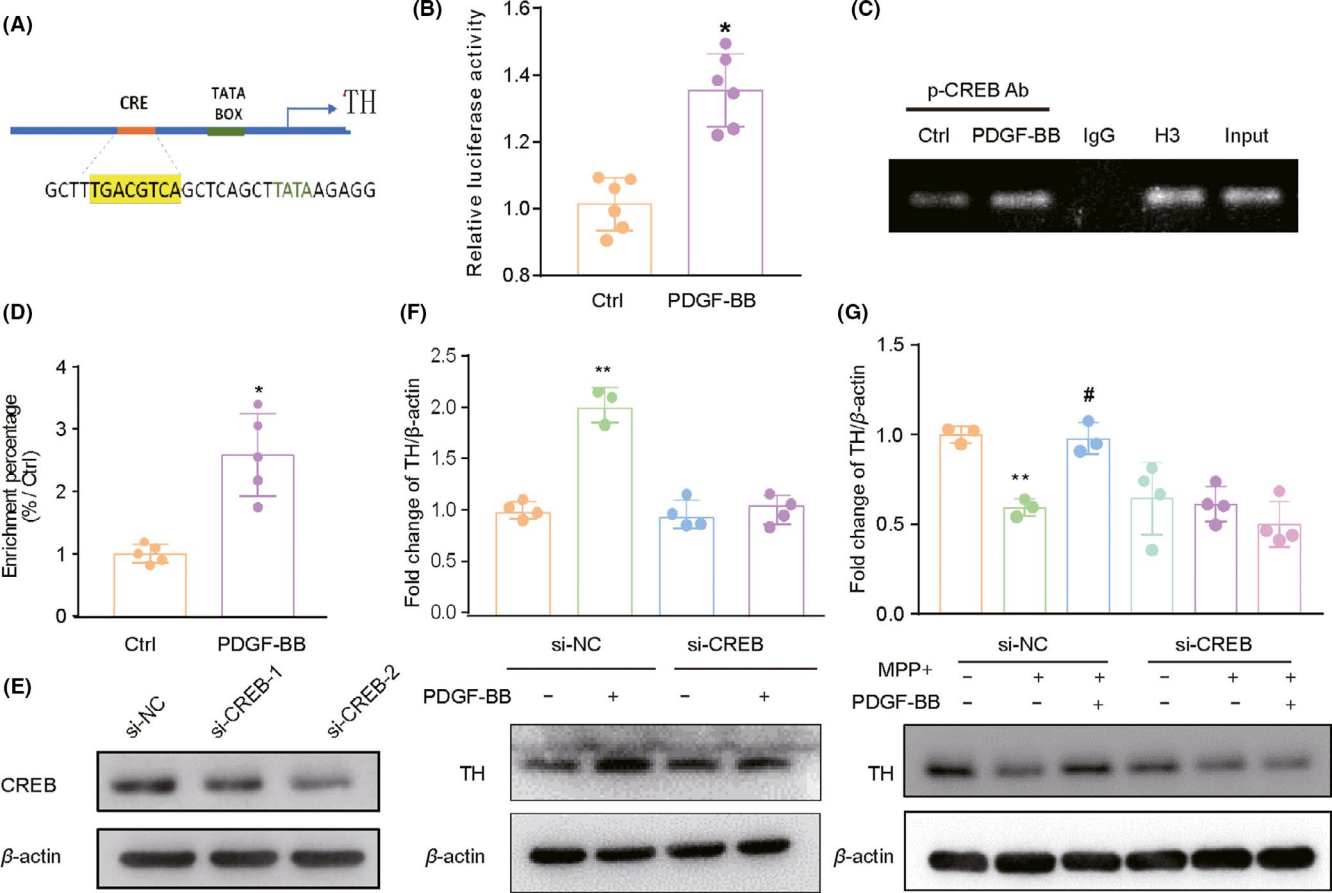
CREB play an essential role in PDGF‐BB‐mediated TH induction. (A) Schematic diagram presents the position of CRE in human TH gene promoter region. (B) Luciferase reporter was transfected in SH‐SY5Y cells, and the luciferase activity was analyzed after PDGF‐BB treatment. (C) SH‐SY5Y cells were treated with PDGF‐BB in serum‐free media, the immunoprecipitated chromatin fragments were amplified by semi‐quantitative and (D) RT‐PCR. (E) SH‐SY5Y cells were transfected with either scrambled siRNA or CREB siRNA, Western blotting results validated the most effective siRNA in the system. (F) The level of TH in the presence of PDGF‐BB is monitored by Western blotting, CREB siRNA but not scrambled siRNA inhibited PDGF‐BB‐mediated induction of TH. (G) In the presence of MPP^+^, CREB siRNA but not scrambled siRNA was able to inhibit PDGF‐BB‐mediated induction of TH in SH‐SY5Y cells. *p < 0.05, **p < 0.01, ***p < 0.001 vs. control group, #*p *< 0.05, ## <0.01 vs. MPP^+^ treated group

The cAMP response element (CRE) is a 30 bp DNA fragment with an 8‐base palindrome structure of TGACGTCA, which mainly presents in the promoters of target genes (Figure 5A). Since CREB can bind to the CRE region and regulate TH gene transcription, we then wanted to demonstrate that CREB was directly involved in the transcription of TH genes. For this, we evaluated the recruitment of CREB to the promoter of TH genes by dual‐luciferase reporter assays and ChIP assay. For dual‐luciferase reporter assays, we inserted the TH promoter fragment amplified from human genomic DNA into the PGL‐3 luciferase reporter. Compared with the control group, the luciferase activities were significantly increased in the PDGF‐BB‐treated group, indicating that PDGF‐BB treatment promotes the activated CREB binding to the promoter region of TH genes (Figure 5B). For the ChIP assay, SH‐SY5Y cells were treated with PDGF‐BB for 60 min, and after immunoprecipitation of PDGF‐BB‐treated cellular chromatin fragments using antibodies against CREB. As evident from the PCR and RT‐PCR results in Figure 5C,D, PDGF‐BB treatment induced the recruitment of CREB to the promoter of TH genes. Thus, our results demonstrated that PDGF‐BB is capable of stimulating the recruitment of CREB to the TH gene promoter in dopaminergic neuronal cells. The role of CREB activation in PDGF‐BB‐induced TH expression was further confirmed by a siRNA approach to knock down the expression of CREB in SH‐SY5Y cells (Figure 5E). Our results showed that knockdown of CREB by using si‐CREB resulted in significant abolishment of PDGF‐BB‐mediated TH induction as shown in Figure 5F. In addition, we also evaluated the functional relevance of CREB in PDGF‐BB‐mediated TH expression in SH‐SY5Y cells in the presence of MPP^+^. As shown in Figure 5G, PDGF‐BB failure to reverse the reduced expression of TH in the CREB knockdown group, indicated that CREB is essential to induce the expression of TH in MPP^+^ impaired dopaminergic neuronal cells. Taken together, these data suggest that a CREB pathway was involved in PDGF‐BB‐mediated TH expression in dopaminergic neuronal cells, and that this pathway also contributed to the PDGF‐BB‐mediated protection of dopaminergic neuronal cells from MPP^+^‐mediated impairment of TH expression.

### 3.6 CREB was activated in PDGF‐BB administrated MPTP‐lesioned mice model

**FIGURE 6:**
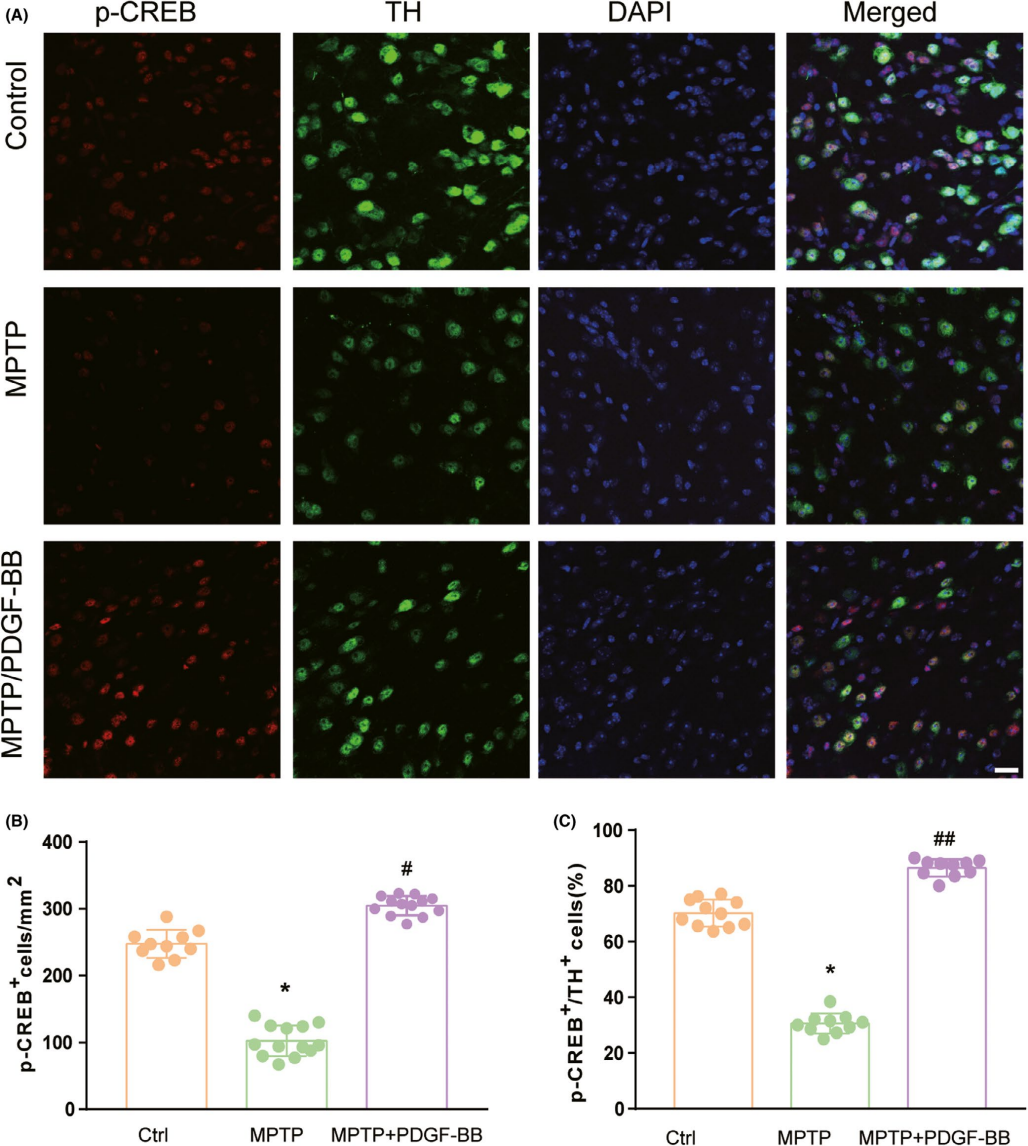
PDGF‐BB exposure results in increased activation of CREB within dopaminergic neurons. (A) Representative image of immunofluorescence staining demonstrating increased p‐CREB in dopaminergic neurons within the SNpc region in PDGF‐BB administrated mice brain. (B) Quantification of p‐CREB and/or (C) TH‐positive cells in the substantia nigra from sections collected from saline, MPTP‐injured, and PDGF‐BB administrated MPTP‐injured groups. All data are presented as mean ± SD of three replicates of indicated treatment conditions *p < 0.05 vs. control group, #*p *< 0.05, ##*p *< 0.01 vs. MPTP group

Since we detected the obvious activation of CREB in our in vitro experiments, we wanted to further validate the activation of CREB in the animal model. To do this, we further examined the expression of p‐CREB and its co‐localization with TH‐positive cells in the SNpc region. As shown in Figure 6A–C, we detected a relatively low signal of p‐CREB in the SN region of the brain sections from saline‐treated mice, while in the MPTP‐lesioned group, the number of immunoreactive cells for p‐CREB was significantly reduced. However, we detected very high levels of p‐CREB in the SNpc region of PDGF‐BB administrated MPTP‐lesioned mice. Since the administration of PDGF‐BB is easy to diffuse to the midbrain, we also collected lower magnification image in that region (Figures S2 and S3). The results between the three groups in the midbrain are similar to that in the SN, which further validated the function of PDGF‐BB on CREB activity in vivo. According to the co‐localization analysis, our results indicated that MPTP administration led to a dramatic degeneration of the dopaminergic neurons in the SNpc, as well as the impaired CREB activation. However, PDGF‐BB administration was able to activate CREB in TH‐positive cells and increases the TH‐immunoreactive intensity in the MPTP‐lesioned group. Thus, we further confirmed the activation of CREB in PDGF‐BB administrated MPTP‐lesioned mice.

## 4 DISCUSSION

Postmortem tissue analysis of PD patients at different stages illustrated that although the number of TH‐positive cells was significantly reduced, visible pigmented neurons remained for up to 10 years in the SN after diagnosis of PD.^4^, ^15^ This observation indicated that, instead of cellular death observed in PD, these cells may remain salvageable. If injured neurons could be supported with certain neurotropic growth factors, those cells could have a potential to exert normal function on neurotransmission. It is well recognized that neurotrophic factors are critical for protection of neurons during cellular injury in the CNS. Over the past several decades, effort has been made to search for efficacious neurotrophic factors that could slow down or even reverse the neurodegenerative process observed in PD and other neurodegenerative diseases.^6^ In the past 10 years, interest has grown in the study of PDGF‐BB, an important neurotrophic factor, and its therapeutic potential in the treatment of PD.

PDGF‐BB belongs to a family of five dimeric ligands, which are assembled from four gene products (PDGF A‐D). The dimerized ligands initiate their function via binding to the receptor tyrosine kinases, PDGF‐αR and PDGF ‐βR. PDGF‐BB was shown to have the highest affinity for PDGFRβ.^16^ Previous studies have indicated that PDGF‐BB play a crucial role in the regulation of neurogenesis in both the developing and adult brains.^17^ The restorative function of PDGF‐BB observed in several PD models was also partially attributed to the improved endogenous neurogenesis elevated upon PDGF‐BB administration.^18^, ^19^ However, besides the great potential to stimulate regeneration, the administration of PDGF‐BB has also been shown to increase the TH+ cell numbers in the SN region of PD animal models. Importantly, the increase in TH+ cells in the SN is not due to proliferation of TH+ cells since neurogenesis in the SN has not been observed so far. Thus, the increase in TH intensity observed in the SN region is probably due to an upregulation in the remaining dopaminergic neurons, and the underlying mechanism is still unclear. MPP+ is a widely used neurotoxin to generate PD model.^20^, ^21^ MPP+ induces DA neuron toxicity by increasing oxidation and mitochondrial dysfunction. The decrease in TH expression is also a remarkable phenomenon in MPP+ exposed DA neurons.^21^, ^22^, ^23^ In our current study, we also used the 1‐methyl‐4‐phenylpyridinium (MPP+) to generate the PD model both in vitro and in vivo. We further confirmed the robust effect of PDGF‐BB on the reversal of TH expression in the MPTP‐lesioned mice model. We also examined the effect of PDGF‐BB in promoting the expression of TH in both primary cultured dopaminergic neurons, as well as SH‐SY5Y cells. Similarly, we also detected that PDGF‐BB treatment was able to reverse MPP+‐induced suppression of TH. These data further support the therapeutic potential of PDGF‐BB in treating patients with early diagnosis of PD.

Through binding to its cognate receptor PDGF‐Rα or PDGF‐Rβ, PDGF‐BB has been shown to activate various signaling pathways, such as PI3K/Akt, phospholipase C (PLC) – γ1, and ERK MAPK, in various cell lines and tissues.^24^, ^25^, ^26^ Akt is a downstream target of PI3K, and both Akt and ERK signaling molecules play essential roles in multiple cellular processes, such as cell proliferation and migration. PDGF‐BB was shown to promote the proliferation and migration of vascular smooth muscle cell through the activation of PDGFRβ/Akt/ERK pathways in several studies.^27^, ^28^ Our previous reports have also demonstrated that PDGF‐BB ameliorated neurotoxin‐mediated impairment of neural progenitor cells (NPCs) proliferation through activation of the ERK MAPK pathway.^29^ In this study, we also detected rapid activation of PI3K/Akt and ERK upon PDGF‐BB treatment in SH‐SY5Y cell. Inhibition of these pathways at a dose that did not affect the basal expression of TH significantly blocked PDGF‐BB‐induced TH expression. These findings thus suggest that activation of PI3K/Akt and ERK MAPK signaling is essential for mediating the restorative effects of PDGF‐BB in dopaminergic neuronal cells.

The transcription factor CREB has emerged as a major regulatory factor for neuronal survival mediated by growth factors such as PDGF‐BB.^30^, ^31^ Our findings also unraveled the key role of CREB in PDGF‐BB‐mediated induction of TH expression in the presence of MPP+. We detected a significant increase in CREB translocation into the nucleus in SH‐SY5Y cells exposed to PDGF‐BB compared with the cells not treated with the growth factor. Further dissection of CREB pathway was done by using the pharmacologic inhibitors, wherein it was identified that the PI3K/Akt and ERK pathway were upstream of CREB and played a critical role in PDGF‐BB‐mediated modulation of TH expression. We also demonstrated that CREB was directly involved in the transcription of TH genes in PDGF‐BB‐treated neuronal cells by ChIP assay. Also, PDGF‐BB has been shown to activate several transcription factors, such as Egr‐1 and AP‐1 to modulate its function.^32^, ^33^ According to relative bioinformatics analysis, the presence of the consensus CRE at the promoter region of the TH gene makes CREB has the greatest potential to be employed for the transcription of TH upon PDGF‐BB exposure. In addition to the upregulation of TH, CREB activation has been involved in other beneficial functions upon PDGF‐BB treatment. Our previous studies demonstrated that CREB is the key regulator for PDGF‐BB‐mediated NPC proliferation.^29^, ^34^ Our earlier studies also demonstrated that activation of CREB is involved in neuroprotection against neurotoxin MPP^+^ induced oxidative stresses. These findings point out that CREB activation might be the key molecular event elicited upon PDGF‐BB application and thus can be considered as an alternative therapeutic target in PD treatment.

In summary, we validated the expressional upregulation of TH in MPTP‐lesion mice upon i.c.v administration of PDGF‐BB for 2 weeks. The in vitro experiments further dissected the involved molecular mechanism in this process. We demonstrated that PDGF‐BB rapidly activated the pro‐survival PI3K/Akt and MERK/ERK signaling pathways, and we also provided the first evidence that PDGF‐BB promoted TH production via activation of the transcription factor CREB, which translocated to the nucleus and bound to the promoter region of TH genes in dopaminergic neuronal cells. Our findings in this study will further support the possibility that targeting PDGF signaling can be harnessed as an adjunctive therapy in PD in the future.

## CONFLICT OF INTEREST

The authors declare no conflict of interest.

## AUTHOR CONTRIBUTIONS

LY and WZY contributed to research concept, research administration, and support. CH and TY carried out the experiments with the help of LZH, CXM, GF, and LYZ. CH and TY performed statistical analyses. LY and CH analyzed the data and wrote the manuscript. All authors edited and approved the final version of the manuscript.

## Supporting information

Fig S1

Fig S2

Fig S3

Fig S4

## Data Availability

The data that support the findings of this study are available from the corresponding author upon reasonable request.
