# Supplementary material for: Platelet‐derived growth factor (PDGF)‐BB protects dopaminergic neurons via activation of Akt/ERK/CREB pathways to upregulate tyrosine hydroxylase
Source: CNS Neurosci Ther. 2021 Aug 4;27(11):1300–12. doi: 10.1111/cns.13708 (PMC8504523; doi:10.1111/cns.13708)
Supplement: Supplementary file 1 — Fig S1 [file CNS-27-1300-s004.pdf]

sFig.1

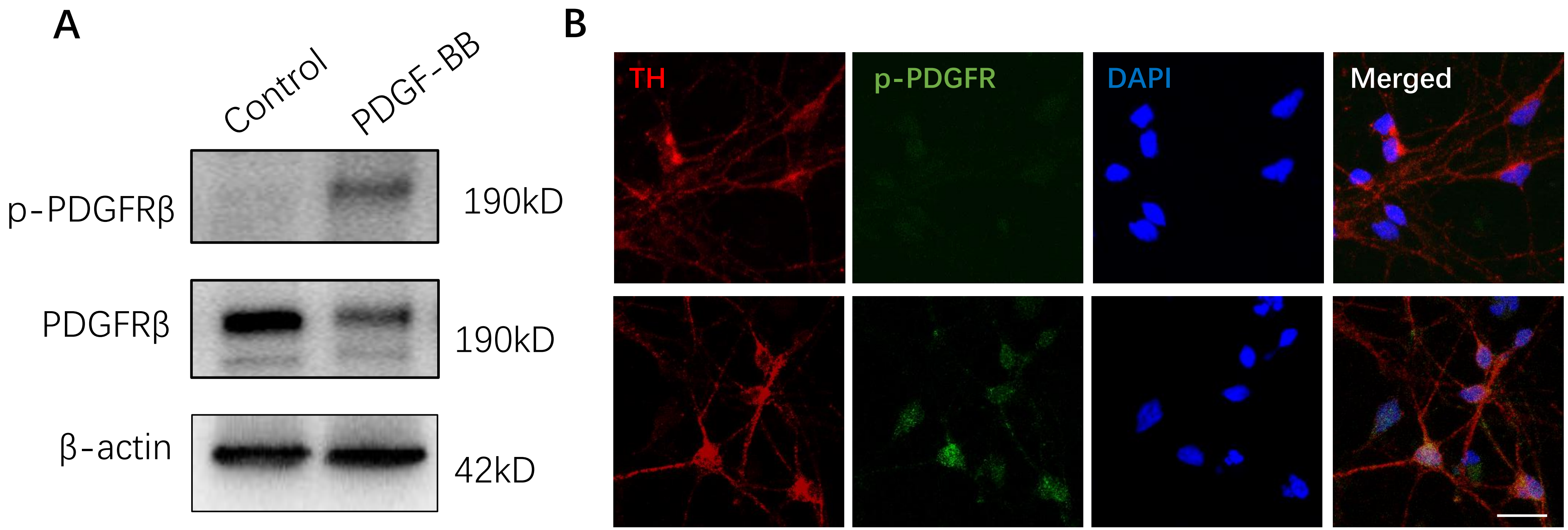

(A) Activation of PDGFRβ was evidenced by increased p-PGFRβ in response to PDGF-BB stimulation followed by WB analysis. **(B)** Primary dopaminergic neurons. Representative immunocytochemical images of control group (upper panels) and PDGF-BB stimulated group (lower panels). The cells were stained for TH (red), p-PDGFR(green) and DAPI (blue), Scale bars, 50 μm.
