# Supplementary material for: Platelet‐derived growth factor (PDGF)‐BB protects dopaminergic neurons via activation of Akt/ERK/CREB pathways to upregulate tyrosine hydroxylase
Source: CNS Neurosci Ther. 2021 Aug 4;27(11):1300–12. doi: 10.1111/cns.13708 (PMC8504523; doi:10.1111/cns.13708)
Supplement: Supplementary file 2 — Fig S2 [file CNS-27-1300-s003.pdf]

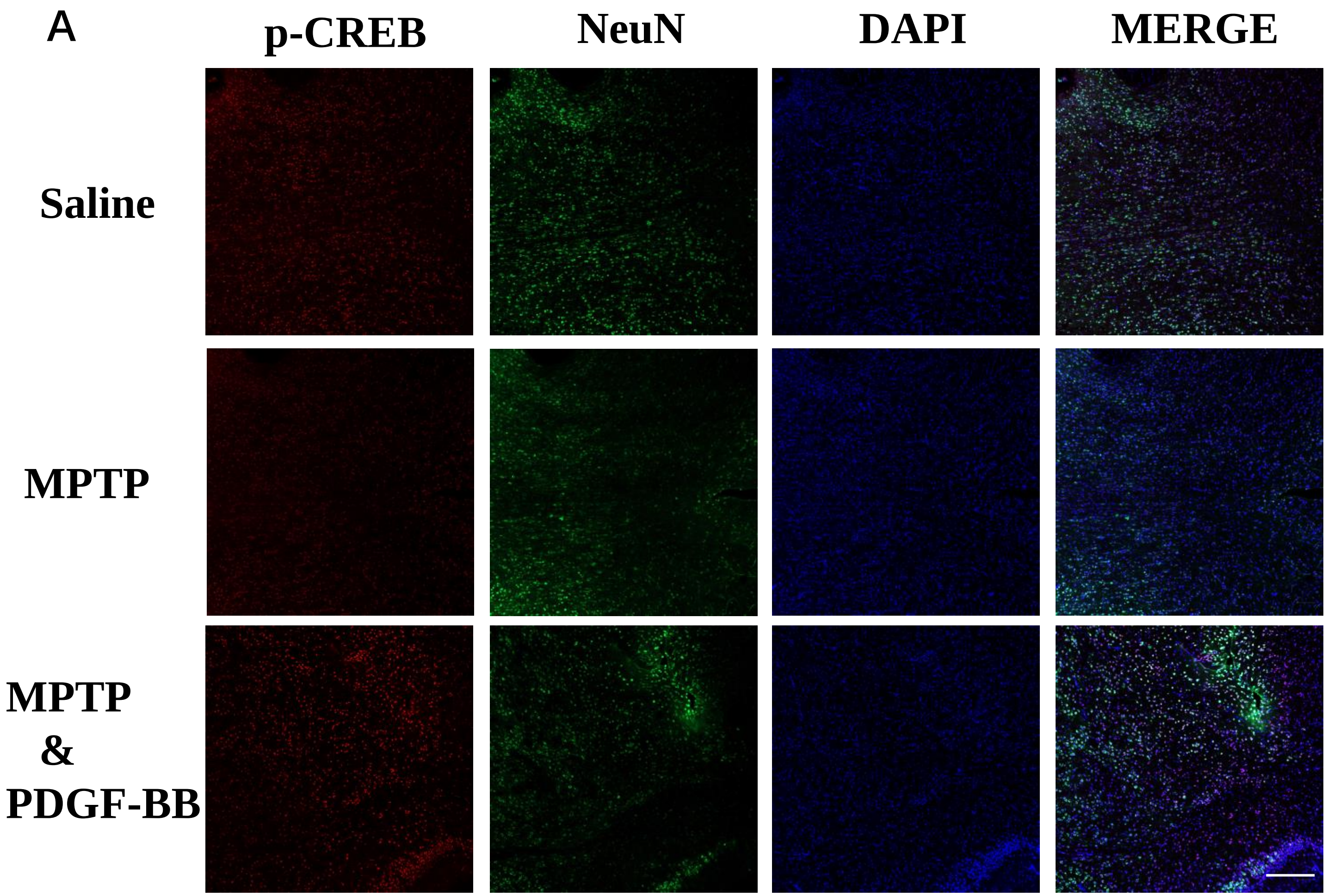


**sFig2.p-CREB in the midbrain:** Representative image of immunofluorescence staining demonstrating increased p-CREB in the midbrain region in PDGF-BB administrated mice brain. The Tiles function on the Zeiss confocal microscopy was used to collect lower magnification image in this region. Scale bar:100μm.
