# Supplementary material for: Platelet‐derived growth factor (PDGF)‐BB protects dopaminergic neurons via activation of Akt/ERK/CREB pathways to upregulate tyrosine hydroxylase
Source: CNS Neurosci Ther. 2021 Aug 4;27(11):1300–12. doi: 10.1111/cns.13708 (PMC8504523; doi:10.1111/cns.13708)
Supplement: Supplementary file 3 — Fig S3 [file CNS-27-1300-s001.pdf]

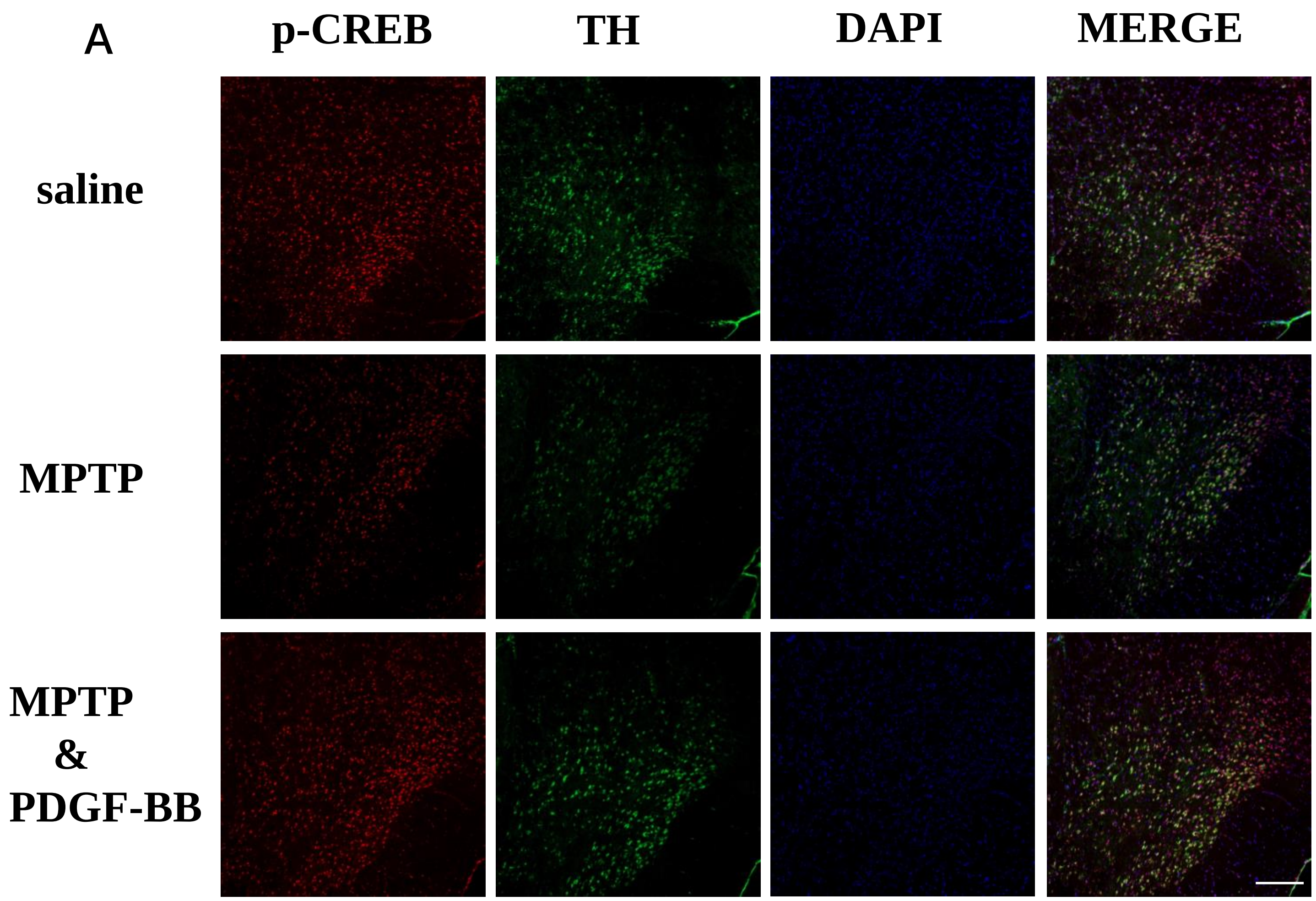

sFig3.p-CREB in the SN region. Representative image of immunofluorescence staining demonstrating increased p-CREB in the SN in PDGF-BB administrated mice brain(lower magnification). Tiles function on the Zeiss confocal microscopy was used to collect lower magnification image in this region. Scale bar:100μm.
