# Supplementary material for: Platelet‐derived growth factor (PDGF)‐BB protects dopaminergic neurons via activation of Akt/ERK/CREB pathways to upregulate tyrosine hydroxylase
Source: CNS Neurosci Ther. 2021 Aug 4;27(11):1300–12. doi: 10.1111/cns.13708 (PMC8504523; doi:10.1111/cns.13708)

sFigure 4

Figure 1H

TH: 58kD

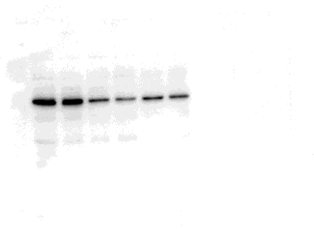

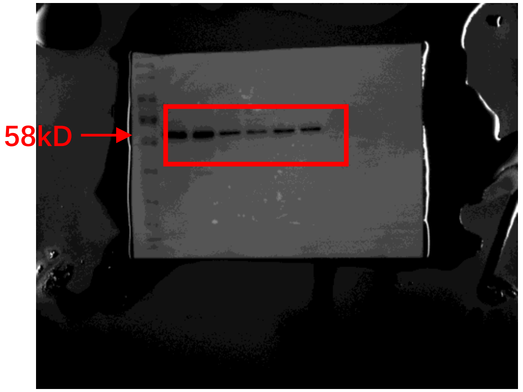

β-actin: 42kD

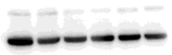

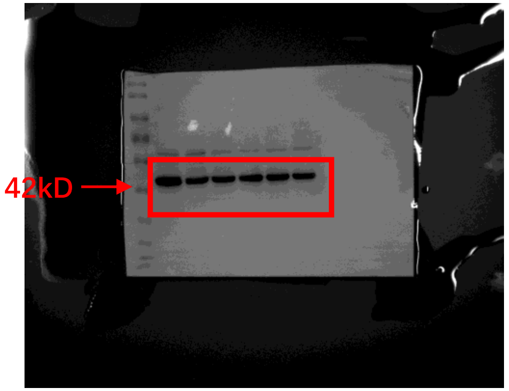

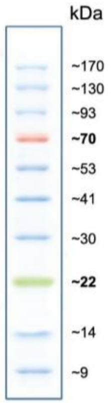

Figure 1J

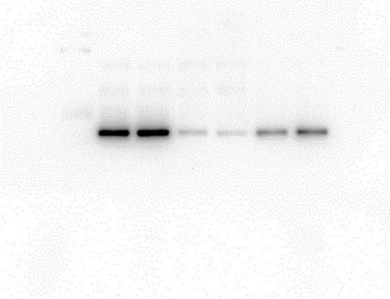

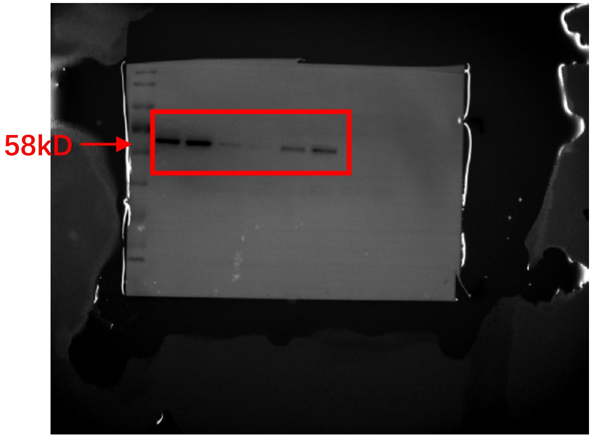

待处理

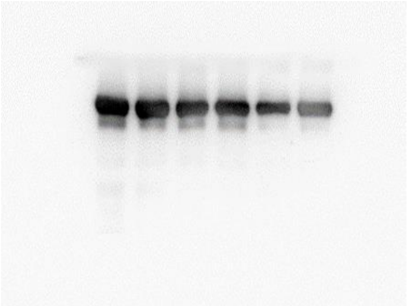

Figure 2B

TH: 58kD

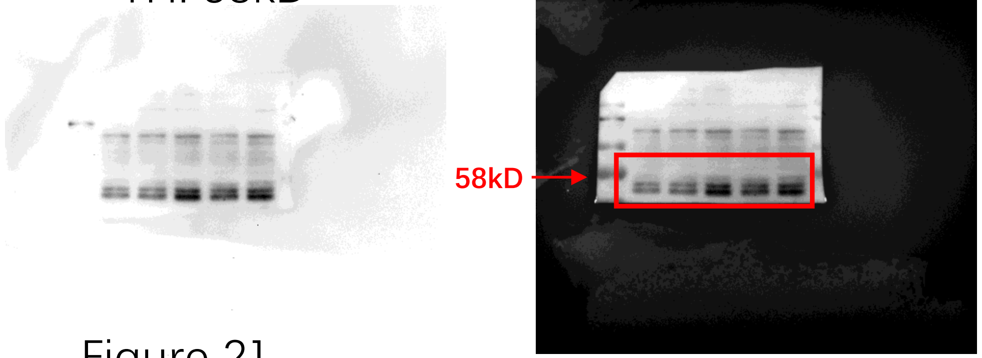

β-actin: 42kD

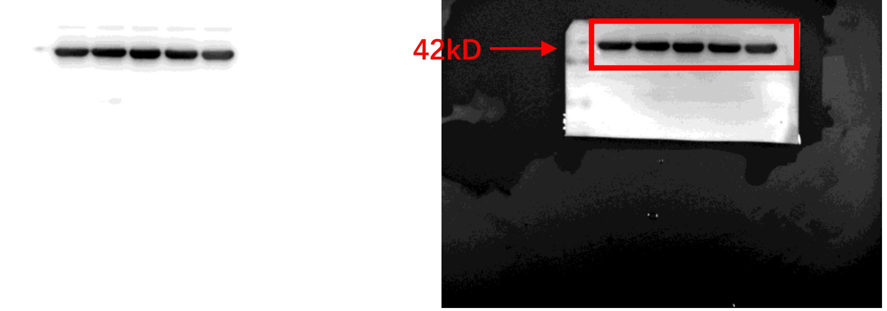

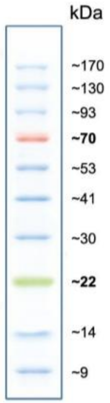

Figure 2J

TH: 58kD

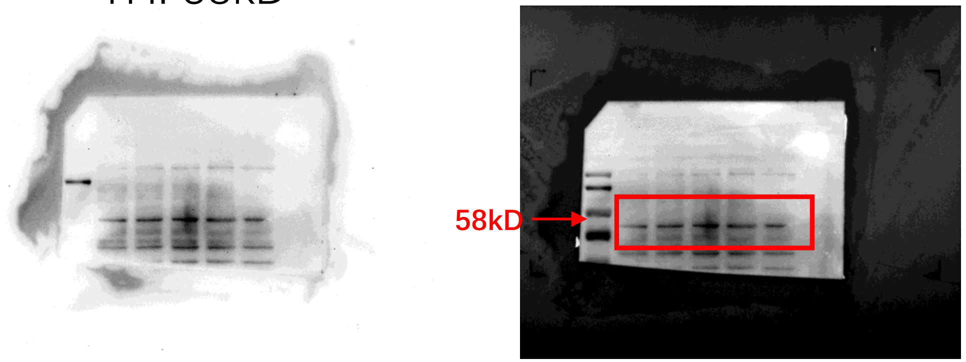

β-actin: 42kD

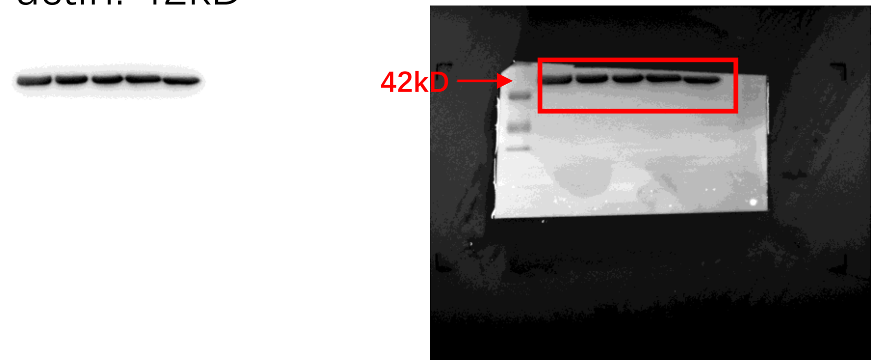

Figure 2F

TH: 58kD

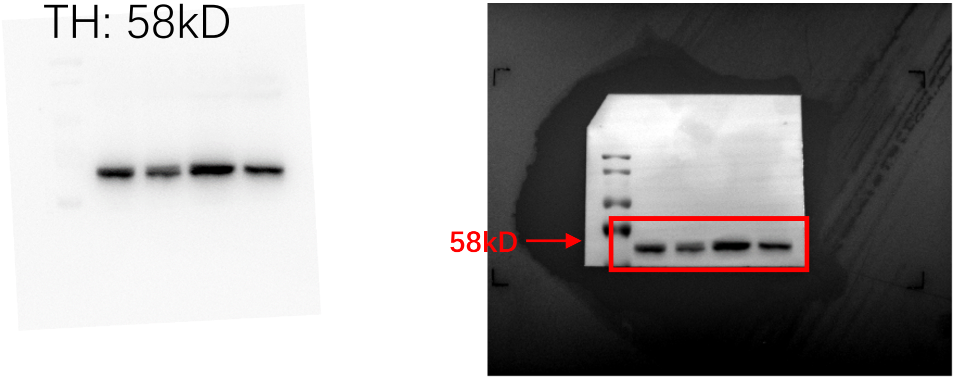

β-actin: 42kD

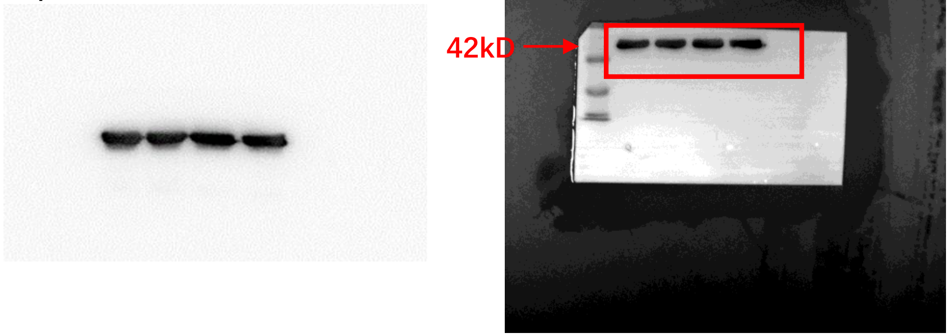

Figure 3A

P-Akt: 60kD

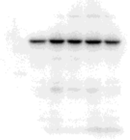

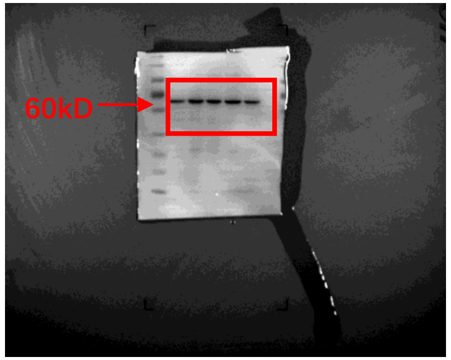

T-Akt: 60kD

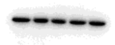

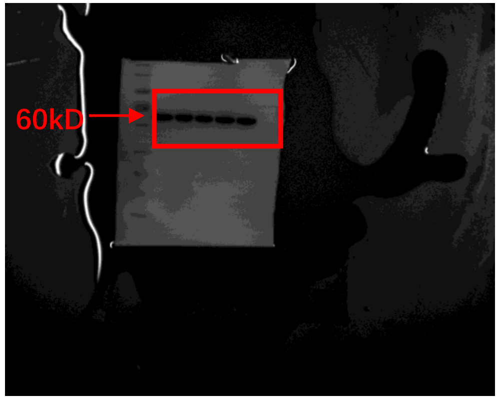

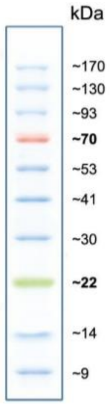

Figure 3B

P-ERK: 42、44kD

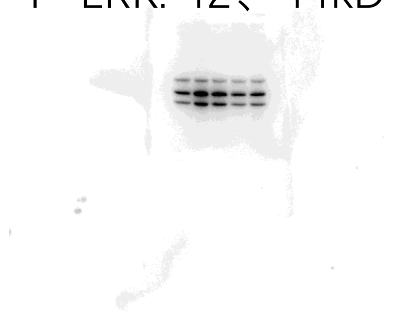

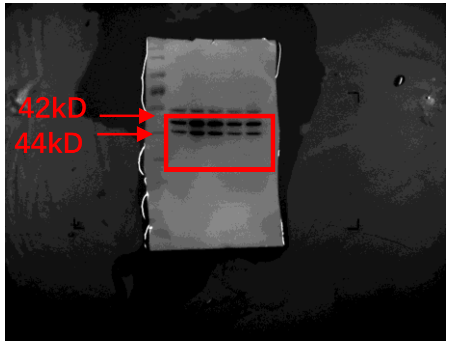

T-ERK: 42、44kD

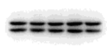

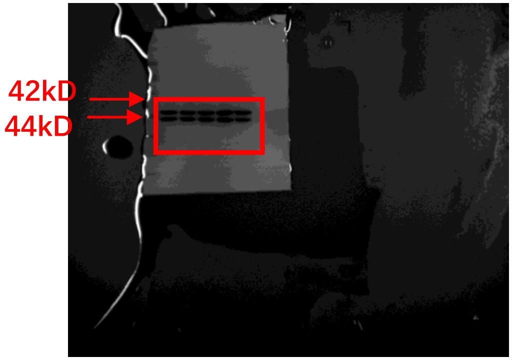

Figure 3C

P-ERK: 42、44kD

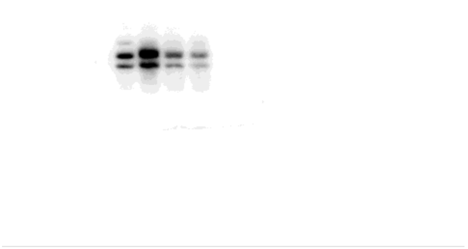

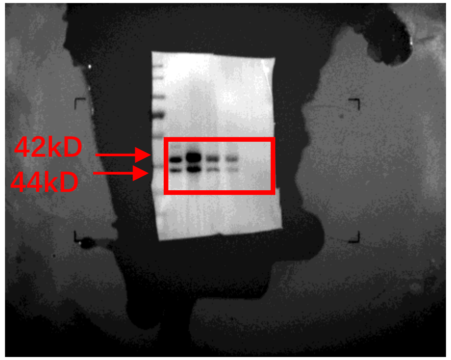

T-ERK: 42、44kD

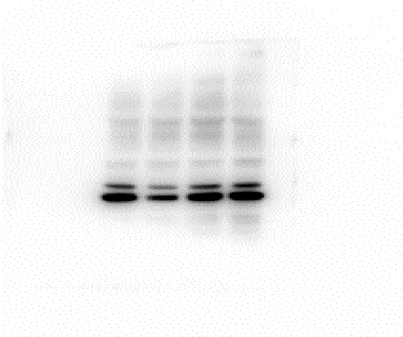

Figure 3D

P-Akt: 60kD

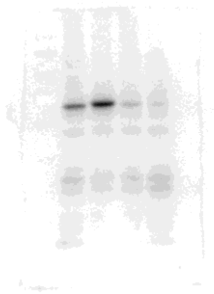

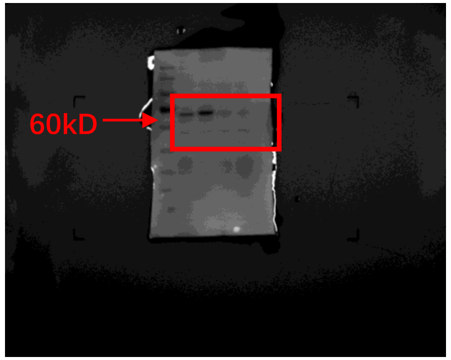

T-Akt: 60kD

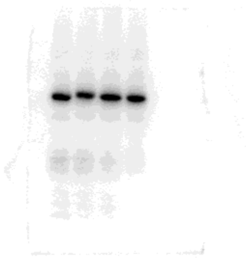

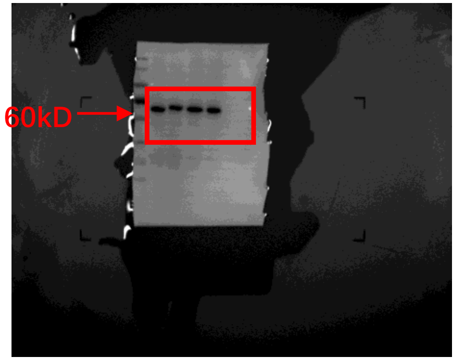

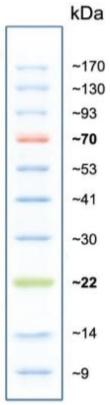

Figure 3E

TH: 58kD

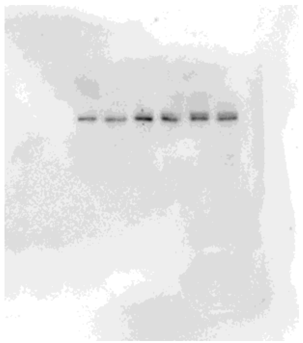

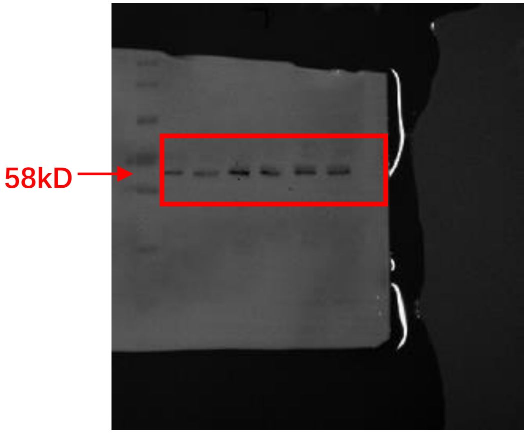

β-actin: 42kD

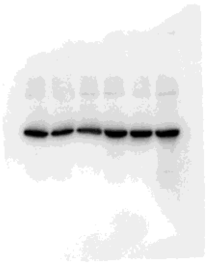

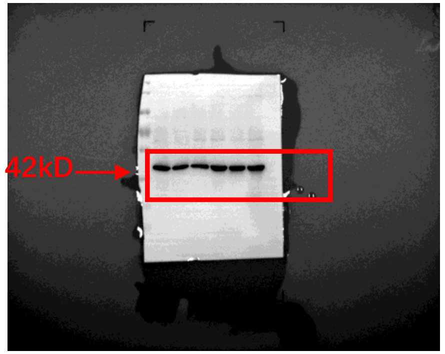

Figure 4A

P-CREB:43kD

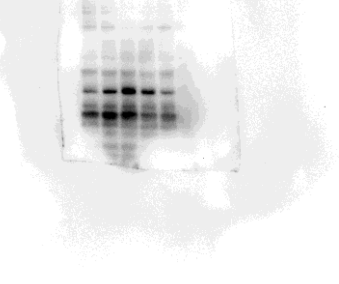

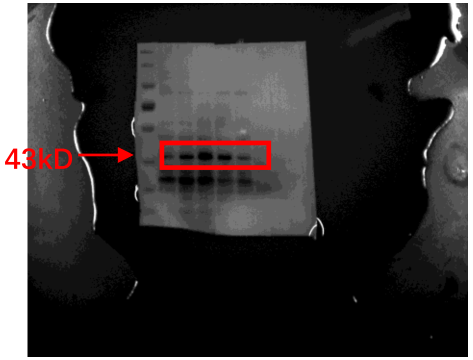

T-CREB:43kD

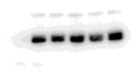

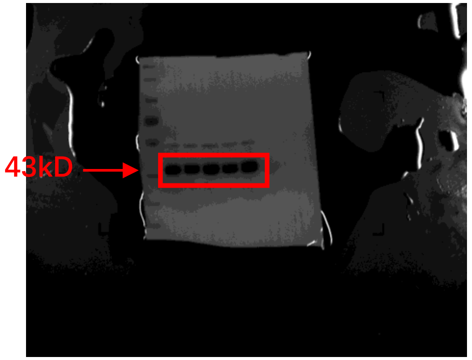

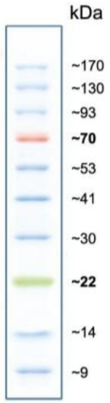

Figure 4B

P-CREB

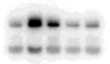

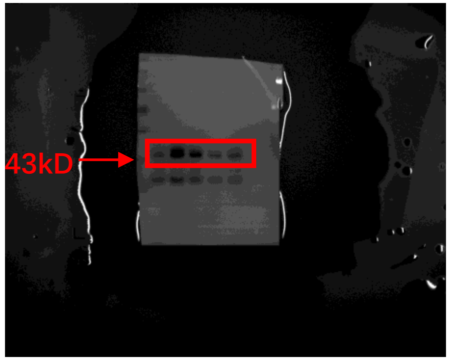

T-CREB

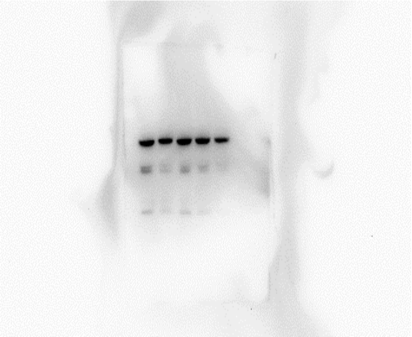

Figure 4E

P-CREB

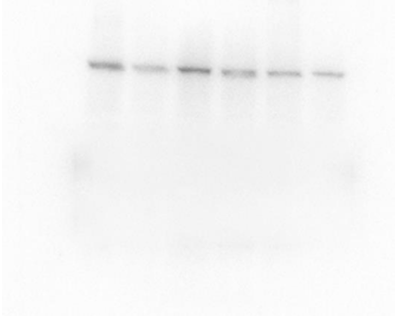

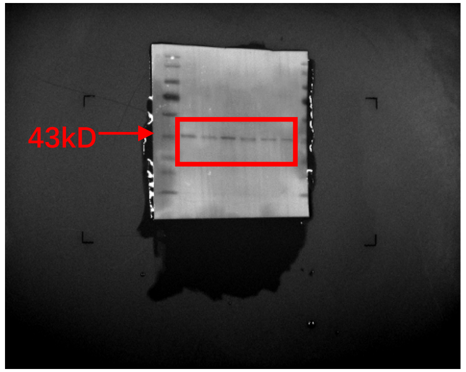

Lamin-B

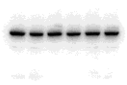

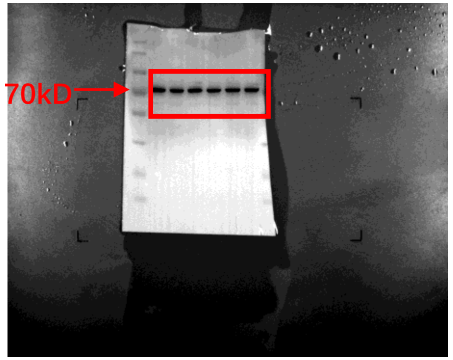

Figure 5C

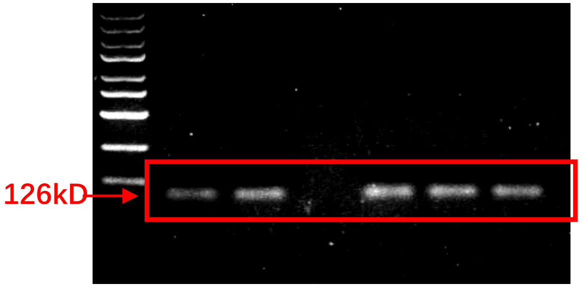

Figure 5E

T-CREB:43kD

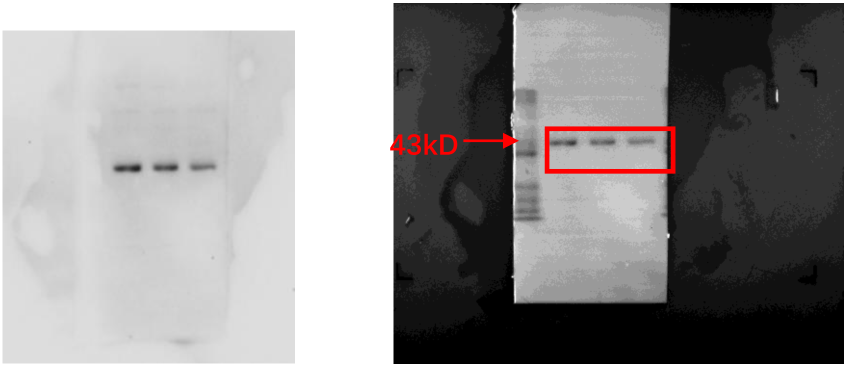

β-actin： 42kD

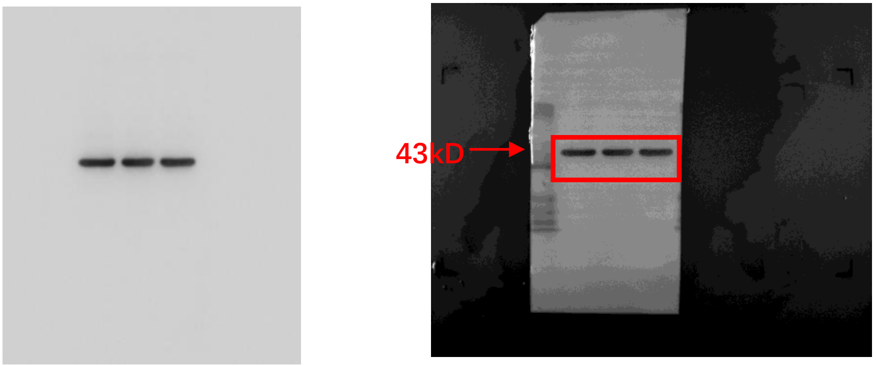

Figure 5F

TH:58kD

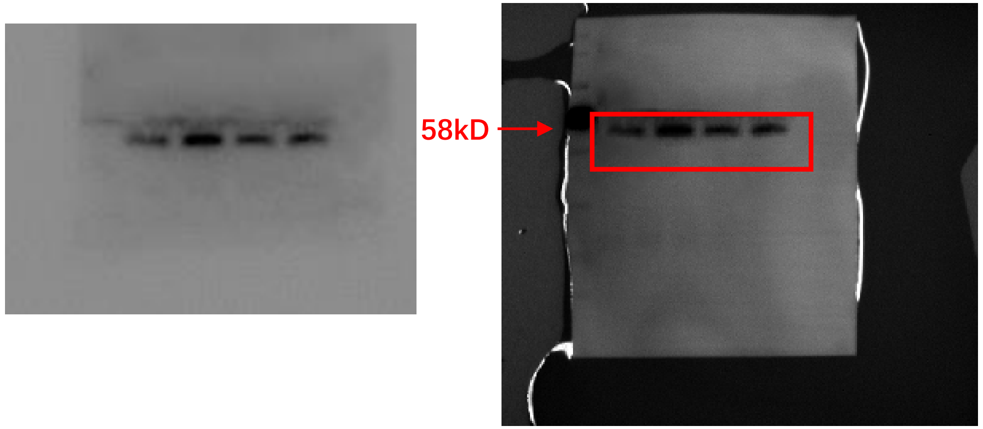

β-actin:42kD

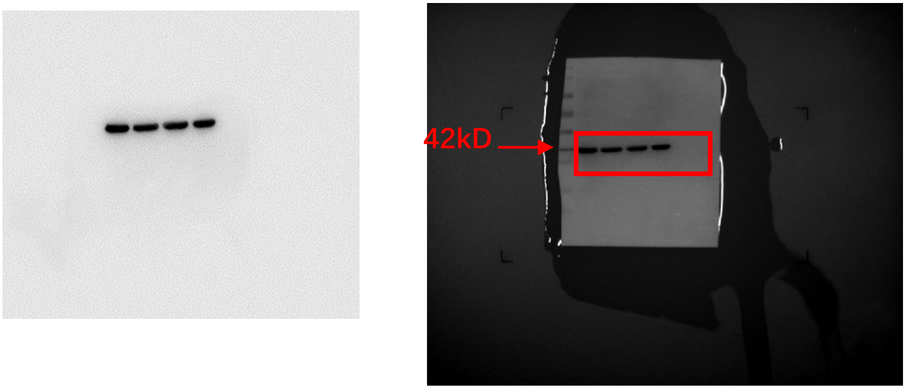

Figure 5G

TH:58kD

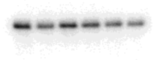

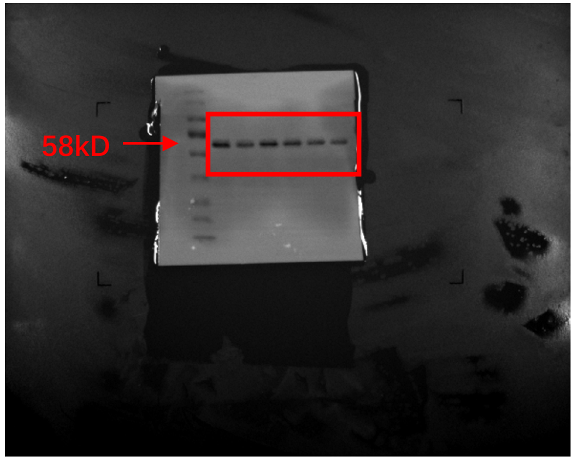

β-actin:42kD

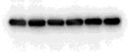

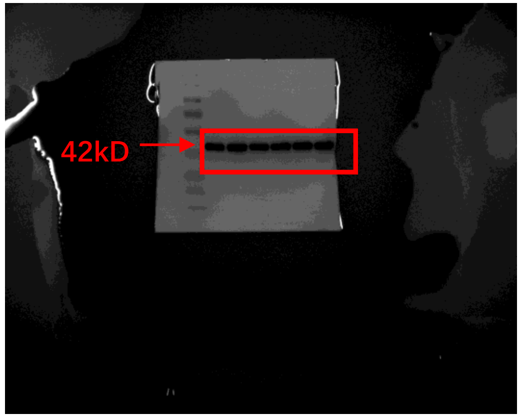

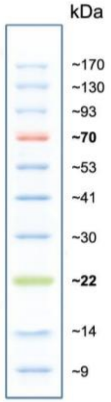

Supplement: Supplementary file 4 — Fig S4 [file CNS-27-1300-s002.pdf]
